# Sex-related differences for uric acid in the prediction of cardiovascular events in essential hypertension. A population prospective study

**DOI:** 10.1186/s12933-023-02006-z

**Published:** 2023-11-01

**Authors:** Maria Perticone, Raffaele Maio, Ermal Shehaj, Simona Gigliotti, Benedetto Caroleo, Edoardo Suraci, Angela Sciacqua, Francesco Andreozzi, Francesco Perticone

**Affiliations:** 1 Department of Medical and Surgical Sciences, University Magna Græcia of Catanzaro, Viale Europa, 88100 Catanzaro, Italy; 2 Geriatric Unit, Azienda Ospedaliero Universitaria R. Dulbecco, Catanzaro, Italy; 3 Cardiology and CICU Unit, Giovanni Paolo II Hospital, Lamezia Terme, Catanzaro Italy; 4 Department of Health Sciences, Magna Graecia University of Catanzaro, Catanzaro, Italy; 5 Internal Medicine Unit, Basso Ionio Hospital, Soverato, Catanzaro Italy; 6 Internal Medicine Unit, Azienda Ospedaliero Universitaria R. Dulbecco, Catanzaro, Italy

**Keywords:** Gender medicine, Uric acid, Cardiovascular risk, Essential hypertension

## Abstract

**Background:**

Uric acid (UA) is an independent prognostic factor for cardiovascular events, but there are no data demonstrating a different risk profile between women and men. Thus, we tested whether UA is associated with a possible sex-related difference in fatal and non-fatal cardiovascular events.

**Methods:**

In this prospective population-based study we enrolled 1,650 never-treated Caucasian hypertensive outpatients referred to Catanzaro University Hospital (Italy). Inclusion criteria were newly diagnosed hypertensive patients, aged 20 years or more. Exclusion criteria were secondary form of hypertension, previous cardiovascular events, rheumatic and non-rheumatic valvular heart disease, prosthetic valves, cardiomyopathies, type-2 diabetes, chronic kidney disease, malignant diseases, gout arthritis and secondary forms of hyperuricemia, liver diseases, peripheral vascular diseases, and heart failure. Anthropometric, clinical, and biochemical parameters were measured. UA prognostic role was investigated by Cox regression analyses. Receiver-operating characteristic curve analyses and area under the curve were used to determine the predictive validity and the optimal cut-off point of UA. We investigated following endpoints: coronary events (fatal and nonfatal myocardial infarction, unstable angina, coronary revascularization procedures, coronary death); fatal and nonfatal stroke; all-cause mortality and major adverse cardiovascular events (MACE).

**Results:**

We enrolled 830 males and 820 females aged 52.2 ± 11.3 years. During 9.5 ± 3.1 years follow-up, there were 424 new clinical events (2.71%): 250 coronary (1.59%), 118 (0.75%) cerebrovascular, and 56 (0.40%) deaths. Comparison between groups demonstrated a higher and significant difference in incidence rate in females for MACE (3.08 vs 2.33%, P = 0.001), coronary (1.82 vs 1.36%, P = 0.014) and cerebrovascular events (0.93 vs 0.57%, P = 0.006). UA at multiple Cox regression analysis resulted a strong and significant predictor of coronary events (HR = 1.493;95% CI 1.375–1.621), cerebrovascular events (HR = 1.256;95% CI 1.109–1.423), MACE (HR = 1.415;95% CI 1.328- 53 1.508), and all-cause mortality (HR = 1.469;95% CI 1.237–1.745) in the whole population and in both groups with a HR higher in females. The best estimated cut-off values of uric acid for males and females predicted these endpoints equally well, but it was always lower in females than males.

**Conclusions:**

We demonstrate, that UA operates with a sex-related impact and best cut-off value in predicting cardiovascular outcomes and all-cause mortality, reflecting a possible sex difference in disease pathophysiology.

## Background

The pathogenetic role of uric acid (UA), the end-product of purine metabolism, in essential hypertension and its natural history have been demonstrated in both experimental and human studies [1–6]. In addition, in the last years, growing evidence has shown the existence of a close association between UA and subclinical organ damage [7–12], as well as with some metabolic alterations such as metabolic syndrome, insulin-resistance and type-2 diabetes mellitus [13–21].

For all these findings, UA has emerged, over the years, as a powerful and independent predictor for cardiovascular events, even after adjustment for other common cardiovascular risk factors, and independently of gout and renal function [22–25]. According with this, we previously demonstrated that the addition of UA, in the normal range, in a model including standard cardiovascular risk factors and estimated glomerular filtration rate (e-GFR), allows to reclassify the global cardiovascular risk of hypertensive patients [26]. The biological plausibility of all these results has been confirmed by the recently issued European Guidelines on the management of arterial hypertension, which have introduced UA among the routine tests for the evaluation of cardiovascular risk of hypertensive patients [27].

At this moment cardiometabolic diseases, despite the commitment of Governments and Scientific Societies, remain the main challenge for the reduction of cardiovascular morbidity and mortality, especially in Western Countries. In this context, growing evidence demonstrate that exists a significant difference for sex-related fatal and non-fatal cardiovascular events, especially in women with type-2 diabetes mellitus [28–30]. Therefore, it remains to be clarified whether a sex-related difference is also present in other clinical contexts to implement a correct therapeutic strategy for preventing fatal and non-fatal cardiovascular outcomes.

For a long time, essential hypertension was considered as the only or, at least, the main biological determinant of the cardiovascular risk profile. In the last decades a different approach for the prevention of cardiovascular events was developed; the actual concept of cardiovascular prevention is based on the recognition and treatment of all the clinical conditions concurring to the definition of global cardiovascular risk. The biological plausibility of this concept is based on the fact that only a small number of hypertensive patients have an elevation of BP alone, with the majority exhibiting additional cardiovascular risk factors, as well as elevated UA values.

Thus, the aim of the present study was to investigate whether the addition of UA—in the normal reference range—to traditional cardiovascular risk factors is associated with a possible sex-related difference in fatal and non-fatal cardiovascular events occurrence in a very large cohort of never-treated and well characterized hypertensive patients.

## Methods

For this population-based prospective study we used data of patients participating to the CATanzaro Metabolic Risk factors (CATAMERI) study. Subjects came to our tertiary University Center directly or were referred by general practitioners for the evaluation of their cardiovascular and/or metabolic risk factors. For the present analysis, from January 2001 until July 2016, we identified 1,650 Caucasian never-treated hypertensive outpatients, 830 men and 820 women, aged 22–72 years (mean ± SD = 52.2 ± 11.3).

Inclusion criteria were newly diagnosed hypertension in both sexes, and an age of 20 years or more. Exclusion criteria were: secondary forms of hypertension detected by a specific protocol, previous cardiovascular events, rheumatic and non-rheumatic valvular heart disease, prosthetic valves, cardiomyopathies, type-2 diabetes mellitus defined as HbA1c ≥ 6.5% or fasting plasma glucose ≥ 126 mg/dl, chronic kidney disease defined by serum creatinine value ≥ 1.5 mg/dl, malignant diseases, gout arthritis and secondary forms of hyperuricemia, liver diseases, peripheral vascular diseases, use of any drugs interfering with UA metabolism (i.e. diuretics, salicylates, cytotoxic drugs, etc.), excessive alcohol consumption defined as having more than 2 drinks (24 g) per day for men and 1 drink (12 g) per day for women and heart failure defined according to both clinical and echocardiographic findings.

The CATAMERI study was submitted and approved on October 17th, 2012 (approval number 2012.63) by Ethics Committee of the Azienda Ospedaliero-Universitaria Mater Domini of Catanzaro (Italy). All investigations were conforming with the principles outlined in the *Declaration of Helsinki*. All the participants gave their informed written consent to study participation.

### Data collection and measures

Data were collected at the first eligibility visit; all patients underwent physical examination, review of their medical history and anthropometric evaluation: weight, height, and body mass index (BMI) expressed as Kg/m^2^. After a preliminary blood pressure (BP) measurement in both arms to exclude a possible difference between them, evaluation of clinic BP was obtained, according with current guidelines at the time of the evaluation [31], after 5 min of quiet rest. A minimum of three BP readings were taken on three separate occasions at least 2 weeks apart. Systolic (SBP) and diastolic (DBP) BP were measured, by a standard validated sphygmomanometer, at the first appearance (phase I) and the disappearance (phase V) of Korotkoff sounds. Baseline BP values represent the average of the last two of the three consecutive measurements obtained at intervals of 3 min. The diagnosis of hypertension was based on values of clinic SBP ≥ 140 and/or DBP ≥ 90 mm Hg, respectively.

Laboratory determinations were performed after a fasting period of at least 12 h. Plasma glucose was determined by the glucose oxidase method (Glucose Analyzer, Beckman Coulter SpA, Milan, Italy), showing an intra-assay coefficient of variation of 2.2% and inter-assay CV of 3.8%. Triglyceride and total, low-density lipoprotein (LDL), and high-density lipoprotein (HDL) cholesterol concentrations were measured by enzymatic methods (Roche Diagnostics GmbH, Mannheim, Germany). Serum creatinine and UA were measured by an automated technique based on the measurement of Jaffe chromogen and by the URICASE/POD method (Boehringer Mannheim, Mannheim, Germany) implemented in an autoanalyzer. For this cohort, values of e-GFR were calculated by using the equation proposed by investigators in the Chronic Kidney Disease Epidemiology (CKD-EPI). We preferred this equation because it is more accurate in subjects with a GFR > 60 mL/min/1.73 m^2^, which our patients were expected to have (creatinine value < 1.5 mg/dL). High-sensitivity C-reactive protein (hs-CRP) was measured by a turbidimetric immunoassay (Behring). Plasma insulin was determined in duplicate by a highly specific radioimmunoassay. Insulin resistance (IR) was estimated by the homeostasis model assessment (HOMA) from the fasting glucose and insulin concentrations according to the equation: HOMA = [insulin (μU/mL _*_ glucose (mmol/L)]/22.5 [32].

All patients, according with specific guidelines, were treated to reduce clinic BP < 140/90 mmHg using standard lifestyle and pharmacological treatment. For this purpose, ACE-inhibitors, angiotensin-II receptor antagonists, calcium channel blockers, diuretics, β-blockers, and α1-blockers were used alone or in combination between them. During the follow-up we planned periodic clinical controls, and a questionnaire was sent to family physicians. All clinical events had to be confirmed by a local Committee based on source data (hospital records, death certificates or other original documents). For this analysis we considered the following clinical events: coronary events (fatal and non-fatal myocardial infarction, unstable angina, coronary revascularization procedures by percutaneous interventions or bypass graft surgery, cardiovascular death or death for any cause, fatal and non-fatal stroke. Diagnosis of acute myocardial infarction was defined according to criteria of the European Society of Cardiology/American College of Cardiology Foundation/American Heart Association/World Heart Federation [33]. Stroke was defined as a new neurological deficit of sudden onset that persisted for at least 24 h [34]. In the analysis we considered major adverse cardiovascular events (MACE), fatal and nonfatal coronary events, fatal and nonfatal stroke, and death for any cause.

### Statistical analysis

Results are reported as mean ± standard deviation (SD), and differences between clinical and biological data were tested by the unpaired Student’s *t*-test and the χ^2^ test for categorical variables as appropriated.

The etiological role of UA levels for explaining the incidence rate of cardiovascular study outcomes, in the whole study population and in males and females separately, was investigated by univariate and multivariate stepwise Cox regression analyses. Tested covariates included UA levels as well as a series of well recognized cardiovascular factors, namely: age, BMI, smoking, total cholesterol, HDL and LDL cholesterol, triglyceride, SBP, HOMA and e-GFR. In the analysis we excluded creatinine to avoid a possible collinearity with e-GFR as well as both fasting glucose and insulin to avoid a possible collinearity with HOMA. In Cox models, data were expressed as hazard ratio (HR), 95% confidence interval (95% CI) and P value.

Event rate is reported as the number of events/100 patient-years based on the ratio of the number of events observed to the total number of patient-years of exposure up to the terminating event or censor. For patients without events, the date of censor was that of the last contact. For the patients who experienced multiple events, survival analysis was restricted to the first event. Survival curves were estimated by use of the Kaplan–Meier product-limit method and compared by using the Mantel log-rank test.

Receiver operating characteristic (ROC) analysis was used to compare the predictive validity, and to determine the optimal cut-off values of UA. Area under the curve (AUC) was also measured to determine the diagnostic power of the test, and to describe the probability that the UA values would correctly identify subjects at risk of cardiovascular events.

All calculations were done by SPSS for Windows Version 20, Chicago, Illinois, USA).

## Results

### Study population

In Table 1 we reported baseline demographic, clinical and biochemical characteristics of the study population stratified by sex. Mean age was 52.2 ± 11.3 years, there were 830 males (50.4%) and 510 (31.0%) smokers. SBP and DBP values were 156.0 ± 11.9 and 93.5 ± 9.3 mmHg, while heart rate was 72.5 ± 9.2 bpm. Metabolic profile was characterized by total cholesterol 207.5 ± 34.1 mg/dl, LDL-cholesterol 124.2 ± 35.3 mg/dl, HDL-cholesterol 49.8 ± 10.9 mg/dl, triglyceride 112.5 ± 27.3 mg/dl, fasting glucose 94.7 ± 10.7 mg/dl, fasting insulin 12.9 ± 3.6 U/L, and HOMA 3.0 ± 0.9. UA mean value was 4.8 ± 1.4 mg/dL; creatinine and e-GFR mean values were 0.96 ± 0.21 mg/dl and 78.6 ± 22.0 ml/min/1.73 m^2^. Mean value of hs-CRP was 4.0 ± 0.9 mg/dl.

**Table 1:** Baseline demographic and clinical characteristics of the study population

	All (n = 1650)	Males (n = 830)	Females (n = 820)	*P*
Age, *yrs*	52.2 ± 11.3	52.3 ± 11.4	52.1 ± 11.2	0.719
Body mass index, *Kg/m*^*2*^	26.9 ± 3.4	27.0 ± 3.3	26.8 ± 3.5	0.232
Current smokers, *%*	510 (31.0)	262 (31.5)	250 (30.5)	0.569
Heart rate, *bpm*	72.5 ± 9.2	72.5 ± 9.3	72.6 ± 9.1	0.825
Systolic BP, *mmHg*	156.0 ± 11.9	155.1 ± 11.7	156.9 ± 12.1	0.002
Diastolic BP, *mmHg*	93.5 ± 9.3	93.2 ± 9.2	93.8 ± 9.3	0.187
Mean BP, *mmHg*	62.5 ± 10.5	61.9 ± 10.4	63.1 ± 10.7	0.021
Total cholesterol, *mg/dl*	207.5 ± 34.1	206.9 ± 32.9	208.1 ± 35.4	0.475
LDL-cholesterol, *mg/dl*	124.2 ± 35.3	123.6 ± 34.2	124.8 ± 36.5	0.491
HDL-cholesterol, *mg/dl*	49.8 ± 10.9	50.1 ± 10.8	49.6 ± 11.0	0.351
Triglyceride, *mg/dl*	112.5 ± 27.3	113.3 ± 27.7	111.6 ± 26.9	0.206
Fasting glucose, *mg/dl*	94.7 ± 10.7	94.5 ± 10.4	95.0 ± 10.1	0.322
Insulin, *U/L*	12.9 ± 3.6	12.5 ± 3.3	13.2 ± 3.9	0.0001
HOMA	3.0 + 0.9	2.9 ± 0.8	3.1 ± 1.0	0.0001
Uric Acid, *mg/dl*	4.8 ± 1.4	4.8 ± 1.3	4.9 ± 1.4	0.133
Creatinine, *mg/dl*	0.96 ± 0.21	0.93 ± 0.21	0.99 ± 0.21	0.0001
e-GFR, *mL/min/1.73m*^*2*^	78.6 ± 22.0	89.1 ± 19.5	67.9 ± 19.0	0.0001
hs-CRP, *mg/dl*	4.0 ± 0.9	3.8 ± 0.8	4.1 ± 0.9	0.001
MACE, *(%)*	424 (2.71)	184 (2.33)	240 (3.08)	0.001
Coronary events, *(%)*	250 (1.59)	108 (1.36)	142 (1.82)	0.014
Cerebrovascular events, *(%)*	118 (0.75)	45 (0.57)	73 (0.93)	0.006
Overall mortality, *(%)*	56 (0.36)	26 (0.33)	30 (0.38)	0.555

Comparing the two groups, we observed that SBP and mean BP, insulin, HOMA, creatinine, and hs-CRP were significantly higher, while e-GFR were significantly lower in females than in males. No significant differences were observed in age, BMI, percentage of smokers, heart rate, DBP, lipid profile and fasting glucose.

### Clinical outcomes

**Fig. 1:**
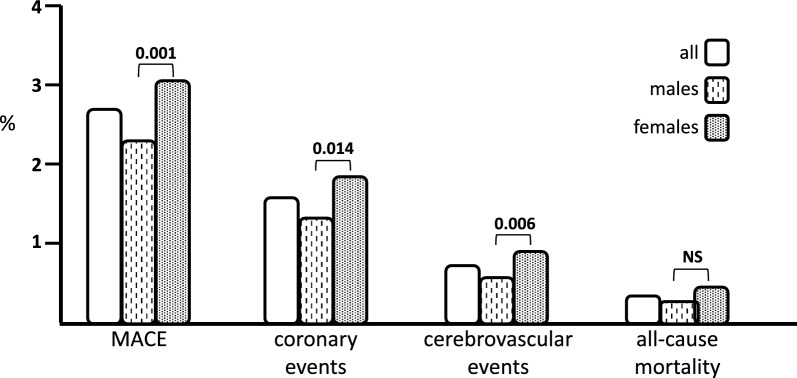
Incidence of cardiovascular events in the study population. We graphically reported the incidence rate (%) of study clinical outcomes occurred in the whole population and in females and females, separately. It is evident a greater and significant incidence in females as compared with males

During a mean follow-up of 9.5 ± 3.1 years, there were 424 new fatal and non-fatal clinical events (2.71%): 250 coronary (1.59%), 118 (0.75%) cerebrovascular, and 56 (0.4%) deaths (Table 1). Interestingly, there was a significant difference between males and females regarding to incidence of MACE (2.33 vs 3.08%, P = 0.001), coronary (1.36 vs 1.82%, P = 0.014) and cerebrovascular events (0.57 vs 0.93%, P = 0.006); while any significant difference was detected in overall mortality (0.32 vs 0.38%; P = 0.555). In Fig. 1 we graphically reported the incidence rate of clinical events in the whole study population and in females and males separately.

### Cox regression analyses: role of UA

On univariate Cox regression analysis, circulating UA levels were significantly related to the incidence rate of all study outcomes (Table 2). On crude Cox analysis in the whole study population, UA (1 mg/dl) was a significant predictor of MACE (HR = 1.433, 95% CI 1.349–1.521), coronary events (HR = 1.499, 95% CI 1.387–1.619), cerebrovascular events (HR = 1.294, 95% CI 1.148–1.458) and overall mortality (HR = 1.431, 95% CI 1.216–1.684). As evident, other independent and strong variables for all prespecified clinical outcomes in the study population, were HOMA and hs-CRP, while age and SBP were retained in predicting both MACE and coronary events. Of interest, with the exclusion for overall mortality, another important and independent prognostic factor for subsequent cardiovascular events resulted the female sex, confirming the existence of a significant biological difference between men and females.

**Table 2:** Univariate Cox regression analysis for incident MACE, coronary and cerebrovascular events, and overall mortality

	MACE			Coronary events			Cerebrovascular events			Overall mortality		
	HR	95% CI	*P*	HR	95% CI	*P*	HR	95% CI	*P*	HR	95% CI	*P*
All												
HOMA, *1 unit*	1.486	1.358–1.626	0.000	1.513	1.347–1.700	0.000	1.503	1.272–1.777	0.000	1.324	1.016–1.725	0.038
Uric acid, *mg/dl*	1.433	1.349–1.521	0.000	1.499	1.387–1.619	0.000	1.294	1.148–1.458	0.000	1.431	1.216–1.684	0.000
Sex, *females*	1.287	1.063–1.559	0.010	1.341	1.044–1.723	0.021	1.446	1.002–2.086	0.049	0.844	0.498–1.429	0.527
hs-CRP, *mg/L*	1.283	1.158–1.422	0.000	1.268	1.108–1.451	0.000	1.276	1.049–1.552	0.015	1.362	1.039–1.786	0.025
SBP, *10 mmHg*	1.139	1.056–1.228	0.000	1.227	1.15–1.350	0.000	1.027	0.885–1.191	0.730	0.990	.0795–1.232	0.925
Age, *10 yrs*	1.010	1.002–1.019	0.016	1.017	1.005–1.028	0.003	1.005	0.989–1.021	0.517	0.994	0.972–1.017	0.601
e-GFR, *10 ml/min/1.7m*^*2*^	0.921	0.882–0.962	0.000	0.992	0.871–0.975	0.000	0.968	0.893–1.049	0.426	0.823	0.725–0.934	0.002
Smoking, *yes/no*	1.185	0.968–1.452	0.101	1.183	0.912–1.534	0.207	1.373	0.947–1.991	0.094	0.890	0.498–1.589	0.694
Total cholesterol, *10 mg/dl*	0.998	0.996–1001	0.286	0.982	0.946–1.018	0.325	0.962	0.912–1.016	0.163	0.977	0.877–1.077	0.590
LDL-cholesterol, *10 mg/dl*	1.001	0.999–1004	0.345	1.014	0.980–1.049	0.416	0.993	0.944–1.044	0.775	1.080	1.008–1.156	0.029
Triglyceride, *10 mg/dl*	1001	0.997–1.004	0.628	0.994	0.950–1.040	0.791	1.037	0.973–1.105	0.261	1.013	0.914–1.123	0.645
Body mass index, *Kg/m*^*2*^	0.979	0.953–1.008	0.157	1.003	0.967–1.040	0.873	0.948	0.898–1.007	0.066	0.922	0.851–1.097	0.069
Heart rate, *10 beats/min*	1.008	0.997–1.018	0.160	1.076	0.940–1.230	0.288	1.140	0.940–1.384	0.184	1.032	0.777–1.369	0.829
Males												
Uric acid, *mg/dl*	1.388	1.259–1.531	0.000	1.447	1.275–1.641	0.000	1.207	1.098–1.477	0.025	1.478	1.165–1.876	0.001
hs-CRP, *mg/L*	1.325	1.142–1.536	0.000	1.285	1.054–1.566	0.013	1.373	1.028–1.833	0.032	1.386	0.978–1.965	0.067
HOMA, *1 unit*	1.291	1.091–1.527	0.003	1.375	1.104–1.713	0.004	1.176	0.845–1.637	0.337	1.193	0.784–1.817	0.410
SBP, *10 mmHg*	1.151	1.026–1.290	0.016	1.328	1.151–1.533	0.000	1.013	0.799–1.285	0.913	0.766	0.544–1.078	0.126
Age, *10 yrs*	1.007	0.995–1.020	0.265	1.018	1.002–1.035	0.030	0.997	0.973–1.021	0.801	0.985	0.955–1.015	0.320
Smoking	1.211	0.908–1.614	0.192	1.124	0.754–1.677	0.565	1.893	1.078–3.325	0.026	0.952	0.438–2.071	0.902
e-GFR, *10 ml/min/1.7m*^*2*^	0.985	0.918–1.058	0.681	1.003	0.913–1.102	0.949	1.067	0.925–1.230	3.73	0.819	0.687–0.977	0.026
Total cholesterol, *10 mg/dl*	0.996	0.924–1.009	0.123	0.961	0.907–1.019	0.181	0.953	0.874–1.040	0.282	1.003	0.903–1.115	0.951
LDL-cholesterol, *10 mg/dl*	1.006	0.967–1.082	0.757	0.995	0.943–1.049	0.839	0.970	0.895–1.051	0.452	1.105	1.007–1.212	0.034
Triglyceride, *10 mg/dl*	1.027	0.975–1082	0.310	1.008	0.941–1.080	0.819	1.132	0.931–1.242	0.196	0.914	0.795–1.051	0.206
Body mass index, *Kg/m*^*2*^	0.973	0.930–1.017	0.224	1.009	0.952–1.068	0.774	0.940	0.860–1.207	0.169	0.897	0.798–1008	0.068
Heart rate, *10 beats/min*	1.120	0.963–1.301	0.141	0.997	0.817–1.216	0.975	1.145	1.008–1.311	0.034	1.156	0.796–1.679	0.446
Females												
HOMA, *1 unit*	1.552	1.395–1.727	0.000	1.541	1.342–1.770	0.000	1.598	1.319–1.936	0.000	1.480	1.048–2.091	0.026
Uric acid, *1 mg/dl*	1.450	1.341–1.567	0.000	1.517	1.373–1.675	0.000	1.323	1.140–1.535	0.000	1.428	1.130–1.803	0.003
hs-CRP, *1 mg/L*	1.223	1.056–1.415	0.007	1.221	1.010–1.475	0.039	1.162	0.885–1.526	−0.281	1.410	0.914–2.176	0.120
SBP, *10 mmHg*	1.106	1.000–1.224	0.049	1.128	0.992–1.284	0.067	1.008	0.831–1.221	0.938	1.262	0.937–1.700	0.126
Age, *10 yrs*	1.015	1.003–1.026	0.015	1.017	1.002–1.032	0.031	1.013	0.992–1.035	0.229	1.006	0.972–1.042	0.730
e-GFR, *10 ml/min/1.7m*^*2*^	0.884	0.822–0.950	0.000	0.885	0.806–0.971	0.010	0.965	0.853–1.093	0.575	0.624	0.464–0.840	0.002
Smoking, *yes/no*	1.127	0.861–1.475	0.385	1.204	0.853–1.698	0.291	1.343	0.875–1.325	0.089	0.842	0.351–2.016	0.699
Total cholesterol, *10 mg/dl*	0.993	0.957–1.030	0.698	0.995	0.949–1.044	0.850	0.968	0.904–1.037	0.360	1.046	0.936–1.069	0.430
LDL-cholesterol, *10 mg/dl*	1.022	0.988–1.058	0.211	1.024	0.980–1.071	0.286	1.005	0.943–1.071	0.870	1.055	0.952–1.170	0.304
Triglyceride, *10 mg/dl*	0.989	0.944–1.036	0.631	0.986	0.929–1.047	0.649	0.971	0.890–1.060	0.512	1.048	0.918–1.196	0.491
Body mass index, *Kg/m*^*2*^	0.985	0.950–1.022	0.421	1.005	0.960–1.052	0.837	0.960	0.897–1.028	0.243	0.943	0.843–1.055	0.308
Heart rate, *10 beats/min*	1.063	0.922–1.225	0.401	1.157	0.964–1.389	0.117	1.163	0.956–1.491	0.125	0.868	0.560–1.346	0.527

The prognostic value of UA on the occurrence of all study outcomes was further tested in multiple stepwise Cox regression models as reported in Table 3. In this Cox model, UA was retained as the independent predictor of all study outcomes; particularly, in the whole study population, 1 mg/dl increase in UA levels provided a significant raise in the risk for MACE (HR = 1.415, 95% CI 1.328–1.508), coronary events (HR = 1.493, 95% CI 1.375–1.621), cerebrovascular events (HR = 1.256, 95% CI 1.109–1.423) and overall mortality (HR = 1.469, 95% CI 1.237–1.745).

**Table 3:** Multivariate Cox regression analysis for incident MACE, coronary and cerebrovascular events, and overall mortality

	MACE			Coronary events			Cerebrovascular events			Overall mortality		
	HR	95% CI	*P*	HR	95% CI	*P*	HR	95% CI	*P*	HR	95% CI	*P*
All												
Uric acid, *mg/dl*	1.415	1.328–1.508	0.000	1.493	1.375–1.621	0.000	1.256	1.109–1.423	0.000	1.469	1.237–1.745	0.000
HOMA, *1 unit*	1.402	1.274–1.542	0.000	1.401	1.239–1.585	0.000	1.469	1.232–1.751	0.000	–	–	–
hs-CRP, *mg/L*	1.302	1.177–1.441	0.000	1.293	1.132–1476	0.000	1.294	1.066–1.571	0.009	1.324	1.010–1.735	0.042
Systolic BP, *10 mmHg*	1.085	1.004–1.172	0.040	1.167	1.057–1.288	0.002	–	–	–	–	–	–
Age, *10 yrs*	1.010	1.002–1.019	0.020	1.015	1.004–1.027	0.007	–	–	–	–	–	–
e-GFR, *10 ml/min/1.7m*^*2*^	–	–	–	–	–	–	–	–	–	0.855	0.755–0.968	0.013
Males												
Uric acid, *mg/dl*	1.372	1.240–1.519	0.000	1.435	1.261–1.633	0.000	1.183	1.086–1.282	0.18	1.388	1.270–1.613	0.000
hs-CRP, *mg/L*	1.277	1.101–1.480	0.001	–	–	–	1.473	1.082–2.005	0.014	–	–	–
HOMA, *1 unit*	1.216	1.029–1.438	0.022	1.270	1.023–1.576	0.030	–	–	–	–	–	–
Systolic BP, *10 mmHg*	–	–	–	1.292	1.113–1.500	0.000	–	–	–	–	–	–
Age, *10 years*	–	–	–	1.017	1.000–1.034	0.050	–	–	–	–	–	–
Heart rate, *10 beats/min*							1.419	1.062–1.896	0.018			
e-GFR, *10 ml/min/1.7m*^*2*^	–	–	–	–	–	–	–	–	–	0.685	0.572–0.821	0.000
Females												
HOMA, *1 unit*	1.526	1.356–1.517	0.000	1.510	1.294–1.763	0.000	1.554	1.270–1.901	0.000	–	–	–
Uric acid, *1 mg/dl*	1.431	1.318–1.553	0.000	1.509	1.360–1.676	0.000	1.273	1.093–1.482	0.002	1.416	1.113–1.800	0.005
hs-CRP, *1 mg/L*	1.367	1.182–1.580	0.000	1.401	1.166–1.683	0.000	–	–	–	–	–	–
e-GFR, *10 ml/min/1.7m*^*2*^	0.915	0.854–0.981	0.012	–	–	–	–	–	–	0.634	0.470–0.854	0.003
Age, *10 years*	–	–	–	1.018	1.002–1.033	0.026	–	–	–	–	–	–

Interestingly, Cox analysis also demonstrated that females, in comparison with males, have an increased risk (+ 28.7%) for the occurrence of MACE, coronary (+ 34.1%) and cerebrovascular (+ 44.6%) events.

### ROC analysis

**Fig. 2:**
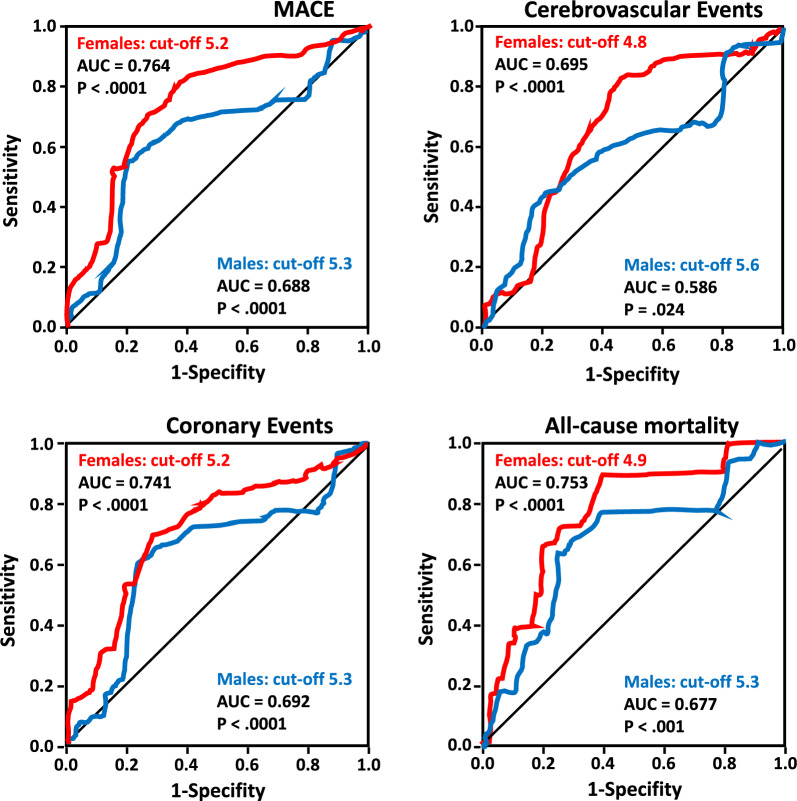
ROC analysis in the study population. Receiver-operating characteristic curves depicting uric acid in predicting all study clinical events in both females and males groups are reported. As evident, the area under the curve (AUC) for all study clinical events was higher in the females group as compared with males group. The best cut-off value of uric acid for cardiovascular events ranges from 4.8 to 5.2 mg/dl in females, while in males group it ranges from 5.3 to 5.6 mg/dl

In Fig. 2 we reported the ROC curves for regression-fitted values of serum UA, in predicting MACE, coronary and cerebrovascular events and overall mortality in both females and men groups. The best estimated cut-off values of UA for males and females predicted these endpoints equally well. Particularly, the best UA cut-off, in the women group, ranges from 4.8 to 5.2 mg/dl, while in the men group its range was from 5.3 to 5.6 mg/dl.

**Fig. 3:**
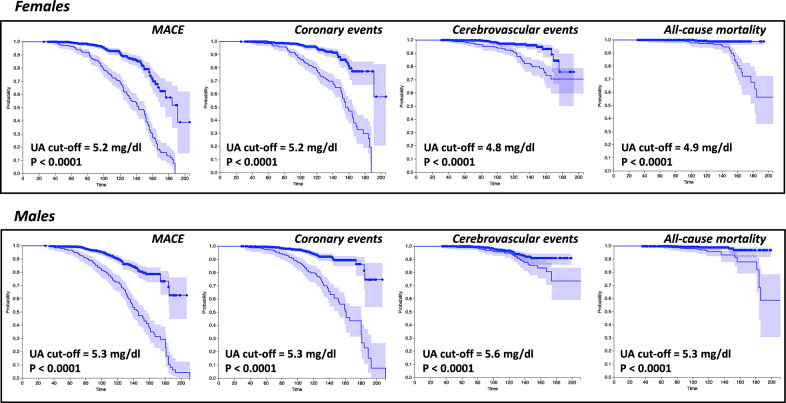
Kaplan–Meier curves for the study population. Kaplan–Meier survival curves for all study clinical outcomes are reported for the study population divided by sex and stratified according to uric acid best cut-off

In Fig. 3 we graphically reported the Kaplan–Meier survival curves, in women and men separately, for each prespecified cardiovascular events in patient’s groups subdivided into above and under best cut-off of serum UA.

In Table 4 we summarized the AUC with 95%CI and the best cut-off serum UA values for all cardiovascular events in the whole study population and in female and male groups separately. As evident, and prognostically relevant, in the females group the AUC values were always lower than in the male group.

**Table 4:** Area under the curve and best cut-off value of serum uric acid for cardiovascular events in the whole population and in both women and men groups

	AUC	95% CI	Best cut-off (mg/dl)
MACE			
Whole population	0.730	0.700–0.760	5.2
Females	0.764		5.2
Males	0.688		5.3
Coronary events			
Whole population	0.710	0.673–0.748	5.2
Females	0.747	0.699–0.79	5.2
Males	0.692	0.634–0.749	5.3
Cerebrovascular events			
Whole population	0.649	0.595–0.703	4.9
Females	0.695	0.612–0.730	4.8
Males	0.586	0.504–6.34	5.6
Overall mortality			
Whole population	0.709	0.639–0.780	4.9
Females	0.753	0.663–0.844	4.9
Males	0.677	0.573–0781	5.3

## Discussion

Our data, obtained in a large and well characterized population of newly diagnosed hypertensive patients, confirm that UA, even if in a normal range, is an independent prognostic factor for cardiovascular events and overall mortality [6, 22–26]. In addition, present data demonstrate that exists an important sex-difference in all cardiovascular outcomes and overall mortality risk associated with serum UA levels as demonstrated by the Cox model, adjusted for a series of potential confounders. In fact, this analysis clearly demonstrated that serum UA is associated with a higher cardiovascular risk, ranging from 30 to 45% as obtained in the univariate Cox analysis, in the group of women compared to that of men for all cardiovascular outcomes and overall mortality. This evidence has an important biological and clinical significance because, for a very long time, the cardiovascular risk was underestimated among females as it was historically recognized as a clinical condition mainly impacting males. Probably, also for these reasons, women have been treated less, affecting a higher cardiovascular morbidity and mortality especially in the post-menopausal period.

The clinical and biological importance of our data is even more relevant since no statistically significant differences in mean UA values, as well as for other hemodynamic and metabolic variables, between the women and men groups have been documented. All this allows us to hypothesize the possible activation by UA of different pathogenetic mechanisms operating in the onset and progression of vascular damage that the present study, obviously, cannot discriminate. However, it is clearly demonstrated that UA has proinflammatory effects [35–37] and is associated with early vascular wall damage, as endothelial dysfunction and intima-media thickness [8, 9, 12, 36], and insulin-resistance and type-2 diabetes mellitus [18–21], all factors that mainly contribute to the appearance and progression of cardiovascular continuum. In this context it is relevant to note that the female group shows significantly higher levels of hs-CRP, insulin and HOMA which, given the above, can help to explain, at least in part, the different risk profile observed between males and females. Of interest, in the present study we have not documented any significant difference between the groups in the BMI which, as known, can affect a different degree of insulin resistance. In keeping with this, Maloberti et al. demonstrated that hypertensive women with hyperuricemia, in comparison with men group, showed a higher prevalence of subclinical target organ damage, in particular renal impairment [38], partly mediated also by metabolic alterations that are associated with hyperuricemia [18–21, 39].

Furthermore, present data and other previous evidence remark the importance to draw the attention of regulatory authorities and healthcare systems to the different risk profile of fatal and non-fatal cardiovascular events [40, 41] existing between men and women and, probably, due also to sex steroids that can affect both sympathetic nervous and renin-angiotensin systems [42]. However, recent evidence continues to demonstrate that, although women have a greater cardiometabolic risk profile, the female gender is associated with lower initiation rates of cardiometabolic protective drugs such as SGLT2 inhibitors and GLP1 agonists [43]. In addition, the same guidelines are less insightful in recommending, again in women, a tighter control of cardiometabolic risk factors; a wrong attitude since the risk for cardiovascular events increases at a lower BP level in females than in males [44].

Nevertheless, certainly not of minor importance is the fact that women have a significant reduction in glomerular filtration rate—as much as 21 ml/min/1.73 m^2^—compared to men irrespective of age and BP values which are the same in the two groups. It is probably that this renal impairment may contribute, at least in part, to the excess risk observed in women; in fact, it is well demonstrated that decline in the renal function is associated with an increased cardiovascular morbidity and mortality in general population and in different setting of patients [45, 46]. According with this, recent experimental findings have demonstrated that UA is able to induce in a rat model a medial vascular thickening of the preglomerular arteriole; interestingly, this primary renal arteriolopathy is BP independent and is due to the activation of the renin-angiotensin system [47]. In addition, the same Authors demonstrated that UA may also directly stimulate vascular smooth muscle cell proliferation in vitro, effect partially inhibited by angiotensin-2 receptors antagonist losartan. The well established uricosuric activity of losartan [48] could explain, at least in part, the reduction of cardiovascular events may be associated to the reduction of UA as observed in the LIFE study and other interventional studies [49–51].

Finally, because the best cut-off of UA for predicting cardiovascular events and overall mortality, according with previously published data [26, 52–55], resulted lower (4.8–5.6 mg/dl) than thatassociated with the risk of gout it could be useful to consider an UA lower diagnostic cut-off to better reclassify cardiovascular risk, as already previously demonstrated by us [26] and recommended by European guidelines for the management of arterial hypertension [27].

In this context, of some interest is the fact that while the best UA cut-off for MACE and coronary events is similar between sexes, it is much higher in women than men for cerebrovascular events and overall mortality. It is not easy to explain this evidence; only hypothetically could it be hypothesized that the greater risk for overall mortality and cerebrovascular events could be supported by the interaction between UA and renal function which, in our population, is significantly lower in women. On the other hand, renal damage is known to be a powerful independent predictor for vascular events and overall mortality [45, 46].

## Conclusions

In conclusion, our data demonstrate, that UA operates with a sex-related impact and a best cut-off value in predicting cardiovascular outcomes and overall mortality, reflecting a possible sex difference in disease pathophysiology and supporting the utility for further investigation to elucidate possible sex-related differences in pathophysiological mechanism of diseases and in pharmacological treatment response. Our findings are concordant with other previously published data [52–55], particularly of that reported in the URRAH study [52], a very large multicentric study performed in a general population, so as to give them an undisputed biological plausibility. Thus, for all these reasons, it is mandatory to design larger clinical trials of UA-lowering strategies in patients with or at high risk of cardiovascular disease to test the optimal cut-off value, as well as the effect in reducing cardiovascular outcomes. If these strategies were shown to be effective in reducing cardiovascular events, they would represent a novel and cost-effective approach in the prevention of cardiovascular diseases.

## Study limitations

Our study has potential limitations because present data were obtained only in Caucasian hypertensives; therefore, should not be applied to other populations. Another limitation of this study consists in the single measurement of UA at baseline, as well as the lack of data regarding the possible increase in UA related to the use/abuse of diuretics. A clear strong point of our work is certainly to be recognized in the longitudinal nature of the study.

## Data Availability

The datasets used and/or analysed during the current study are available from the corresponding author on reasonable request.

## Abbreviations

UA: Uric acid
e-GFR: Estimated-glomerular filtration rate
HbA1c: Glycated hemoglobin
BP: Blood pressure
SBP: Systolic blood pressure
DBP: Diastolic blood pressure
LDL: Low density lipoprotein
HDL: High density lipoprotein
hs-CRP: High sensitivity C reactive protein
IR: Insulin resistance
HOMA: Homeostasis model assessment
ACE: Angiotensin converting enzyme
MACE: Major adverse cardiovascular events
ROC: Receiver operating characteristic
AUC: Area under the curve
HR: Hazard ratio
CI: Confidence interval
